# The Joint Association of Veteran and Post-Traumatic Stress Disorder on Risk of Cardiovascular Diseases

**DOI:** 10.1093/geroni/igaf122.627

**Published:** 2025-12-31

**Authors:** Andrew London, Jacob DeBlois, Kevin Heffernan

**Affiliations:** Syracuse University, Maxwell School of Citizenship and Public Affairs, Syracuse, New York, United States; University of Rhode Island, Kingston, Rhode Island, United States; Columbia University, Teachers College, New York, New York, United States

## Abstract

Identifying subpopulations at elevated risk of cardiovascular disease (CVD) is important for preventive purposes. We use pooled data from the 2021-2023 National Wellbeing Survey (NWS) (N = 17,257) to examine the joint influence of veteran and post-traumatic stress disorder (PTSD) statuses on high blood pressure, high cholesterol, diabetes, and heart disease diagnosis by a health care professional among working-age adults. The NWS uniquely measures PTSD among veterans and non-veterans. In well-controlled multivariable logistic regression analyses, relative to non-veterans without PTSD (henceforth the reference group), the odds of high blood pressure are significantly higher among non-veterans (adjusted odds ratio [AOR]=1.48, p<.001) and veterans with PTSD (AOR=1.63, p<.01), and post-hoc tests indicate that both of these groups also have significantly higher odds of high blood pressure than veterans without PTSD. For high cholesterol, the odds are significantly higher among non-veterans with PTSD (AOR=1.33, p<.001) and both veterans without (AOR=1.24, p<.05) and with PTSD (AOR=1.51, p<.01) relative to the reference group. For diabetes, both veterans without (AOR=1.36, p<.01) and with PTSD (1.72, p<.010) have higher odds relative to the reference group, and veterans with PTSD have significantly higher odds than non-veterans with PTSD. For heart disease, the odds are higher for non-veterans with PTSD (AOR=1.90, p<.001), and both veterans without (AOR=1.38, p<.05) and with PTSD (AOR=2.44, p<.001) relative to the reference group, and veterans with PTSD have significantly higher odds than veterans without PTSD. These results suggest that both veteran and PTSD statuses contribute to CVD risk, and that their influences are at times synergistic.

